# Challenges in the surveillance and control of mosquito-borne diseases in Europe and United States. The perspective from public health experts.

**DOI:** 10.1016/j.onehlt.2025.101133

**Published:** 2025-07-08

**Authors:** Luigi Sedda, Elizabeth Wrench, Thomas C. Moore, Kaitlyn Wolfe, Julie-Anne A. Tangena, Heidi E. Brown

**Affiliations:** a Lancaster Ecology and Epidemiology Group, Lancaster Medical School, Lancaster University, Health Innovation One, Sir John Fisher Drive, LA1 4AT Lancaster, UK; b The University of Arizona, Department of Epidemiology and Biostatistics, Mel and Enid Zuckerman College of Public Health, 1295 N Martin Ave, Tucson, AZ 85724, USA; c Vector Department, Liverpool School of Tropical Medicine, Pembroke Pl, Liverpool L3 5QA, UK

**Keywords:** Mosquito-borne disease policy, Public health, Emerging diseases, Zoonosis, Trapping, Insecticide interventions

## Abstract

The complexity of mosquito-borne diseases and the necessity for cross-sector collaboration present significant challenges, requiring changes in laws, policies, and inter-agency agreements. In this qualitative study we purposively selected and interviewed public health managers from the European Union and United States involved in vector-borne disease surveillance and control and asked them about the barriers currently faced when engaging in their activities. The interviewees highlighted the differences in surveillance and control guidelines between the European Centre for Disease Prevention and Control and the US Centers for Disease Control and Prevention which reflect structural political differences between the European Union and United States. The lack of centralisation increases uncertainty in applying mosquito surveillance and control guidance. In addition, limited resources and modelling capabilities hinder effective surveillance and control. The public health agents recognised that community engagement and transparent communication are critical for gaining public support and to succeed in interventions, thus recognition of the values of these collaborations need to be accounted for in disease preparedness. Effective mosquito surveillance and control requires strong organisational bases, coordination among stakeholders, and sufficient resources, as advocate by one health frameworks. Addressing these challenges is urgent due to global trends like climate change and increased international travel, which may heighten the risk of mosquito-borne disease outbreaks.

## 1 Introduction

Emerging and re-emerging vector-borne diseases remain a challenge for disease control and prevention [1]. The preliminary and concurrent task in control is surveillance, which is not restricted to identify circulating vectors and pathogens, but also to identify emerging threats, predict transmission, establish capacity and response, and modelling to minimize pathways to introduction [2]. Funding to maintain surveillance capacity fluctuates with the sporadic emergence of vector borne diseases having detrimental effects on preparedness and response [3]. The importance of sustained and reliable surveillance becomes even more critical as climate change shifts baselines, moving populations risk importing pathogens or creating hotbeds of transmission when crowding occurs, and both legal and illegal trade practices establishing new pathways for disease spread [4].

Among various disease vectors, mosquito-borne diseases continue to represent a significant burden globally. They account for 17 % of all infectious diseases yielding an estimated one million deaths each year [5]. An estimated 80 % of the global population is at risk from health and economic impacts of vector-borne diseases, primarily mosquito-borne [6]. This burden is expected to grow with climate change driven expanded disease ranges and shifts in mosquito behaviour and pathogen-host interactions (for viruses see [7]).

The goal of this study is to identify the major challenges in the surveillance and control of established or emergent mosquito-borne diseases in the European Union (EU) and United States (US) by interviewing public health managers/experts from different institutions in EU countries and US states. By doing so we aim to promote a debate toward potential solutions to current operational gaps in mosquito surveillance and control.

## 2 Material and methods

### 2.1 Recruitment

To identify suitable interviewees, we focused on professionals working within public health, academia, or research institutions who were actively engaged in mosquito control initiatives. In the United States, we employed two main approaches to select participants. First, potential jurisdictions were drawn from a systematic review of West Nile Virus (WNV) in the US conducted by [8] which highlighted 24 studies involving 48 co-authors affiliated with 17 health departments or mosquito abatement districts in nine states (Arizona, California, Colorado, Connecticut, Illinois, Louisiana, New York, South Dakota, and Texas). Second, in order to ensure adequate representation from southern states we conducted an online search to identify mosquito control agencies in Alabama, Louisiana, New Mexico, and Florida. This process led to the identification of 11 agencies, from which we obtained contact information (such as organisational emails or social media accounts like LinkedIn) through publicly accessible sources.

In the EU, individuals were selected from the European Centre for Disease Control and Prevention (ECDC), VectorNet [9] website contacts, and author list extracted from [10]. VectorNet is a European project funded by ECDC and European Food Safety Authority that aims to improve preparedness and response for vector-borne diseases, and supports the collection of data on vectors and pathogens related to both animal and human diseases. It is composed by public and animal health agencies encompassing EU, European Economic Area, and EU Enlargement policy and European Neighbourhood Policy partner countries, although for this study we only considered EU countries. Additional contacts were added when provided by other interviewees (snowball sampling).

Individuals were contacted up to three times between June 21st to November 13th, 2023, or until a response was received from a suitable representative of the targeted country or organisation. In the US, among the 24 agencies identified through published literature, nine failed to respond, five declined, one redirected us to a colleague who also declined, and nine interviews were ultimately conducted. Of the 11 agencies identified from southern states, three agreed to participate, two declined, and six did not respond; resulting in 11 interviews (30.6 %). Fourteen of the 29 European contacts initially identified, were interviewed (48.3 %). Five declined to be interviewed (busy or no longer in vector control), eight never returned contact and one returned contact too late in the project. The fourteen EU interviewees were from Belgium, Denmark, France, Finland, Germany, Italy, Netherlands, Romania, Spain, Sweden, Switzerland, and United Kingdom.

### 2.2 Ethics

No ethics approval was necessary as the project was considered an evaluation and not human subjects research by both Lancaster University and The University of Arizona institutional ethics review boards.

### 2.3 Interviews

Prior to the interviews, an interview guide was developed with a list of questions and topics to explore (Appendix A). Participants were asked to agree to the recording and transcription at the beginning of the interview and reminded that they could terminate the interview, or the use of their interview information, at any time. By using Teams (Microsoft Corp., Redmond, WA) or Zoom (Zoom Video Communications, Inc., San Jose, CA), interviews were conducted remotely, transcribed, transcriptions cleaned and imported into NVivo (Lumivero) for coding. Once transcribed, the videos were deleted.

### 2.4 Analysis

We used grounded theory approach to group data into categories and themes [11], using both deductive and inductive coding. Deductive codes were developed based on the research question ‘what are the current operational gaps in the surveillance and control of vector-borne infectious diseases in the EU and US?’ including challenges to statistical model uptake, use of apps in analysis and communication, successes and failures in surveillance and control, and future vision. Inductive coding based on the initial review by authors added use of alerts, capacity and collaboration, dissemination and engagement, and seasonality. The EU team at Lancaster University met to identify candidate codes, which were shared with the US team (based at the University of Arizona) to identify additional codes. Each transcript was independently coded by two investigators, usually one of whom had conducted the interview. At the onset, the two teams' coding were compared and refined. After all transcripts were processed, the two principal investigators (LS, HEB) discussed connections in order to identify an agreed upon set of thematic codes.

## 3 Results and discussions

According to the interviewees, the complexity of mosquito-borne diseases and the challenge to working across sectors can be a stumbling block needing changes in laws, policies, and sharing agreements, even within governmental agencies. This challenge also bleeds into how to disseminate the complexity of mosquito-borne disease control messaging to the public [12], speciation (countering the feeling that all mosquitoes are the same and all are efficient vector for diseases) [13], and the lack of disease in some areas that can undermine control efforts, in addition to the “anti-science movement” [14]. Within these themes, most of the interviewees highlighted that the lack of capacity for surveillance data modelling, combined with uncertainty on the surveillance national guidance and lack of centralised efforts, are the major barriers for effective vector-borne disease surveillance and control today (as also highlighted in literature, see for example [15]).

### 3.1 The challenges regarding decentralised surveillance

The US has a long history of control of invasive mosquitoes including *Aedes aegypti* and *Aedes albopictus*, both vectors of mosquito-borne diseases such as chikungunya, dengue, WNV and Zika [16]. Although, even then, efforts and funding fluctuated dramatically with perceived risk [17]. In contrast, it is only the early 21st century incursions of invasive mosquitoes that prompted the establishment of monitoring for invasive mosquito species [18] as part of routine surveillance in most EU countries [19]. In response of the emergence of new mosquitoes and pathogens, ECDC provided guidance on surveillance [20] and weekly updates on the spread of these invasive species [21]. Surveillance for invasive species was reinforced in port of entries, major road networks and migratory sites (for insects and hosts, such as birds) [22].

Despite ECDC being trans-national and the US Centers for Disease Control and Prevention (CDC) being national, they work in a similar capacity, setting surveillance guidelines [20,23] and promoting interventions including awareness campaigns towards physicians, support of laboratory confirmation, active laboratory-based surveillance, case investigation and daily dissemination of information to national and local stakeholders [24,25]. For surveillance and control of nuisance mosquitoes, as well as mosquito-borne disease invasive species, surveillance activities are decentralised to local authorities (authorities loosely defined here to mean national or state health organisations, and private vector control companies) in the EU [26] and in the US [3]. National public health and governmental organisations usually act as coordination centres providing recommendations, evaluations, assessments and alerts:

*…control of mosquitoes is a responsibility of local authorities. So [the public health government agency] provides technical advice on the control, but the actual response would be a local authority response. [EU07]*

*…most of the mosquito surveillance and control efforts are really conducted more at the municipal level, and then they share those results with us here at the County Public Health, and then we take a look at the vector index and which traps were positive and numbers that we're seeing. […] So we're almost like a consultant role rather than being the ones that are actually out doing the trapping and testing and so forth. [US12]*

*So we have a good network to get [mosquito] information, but one of the routine work is to check whether something happens [mostly from literature], whether there is a risk to appear, and then to answer the questions from the agencies or Ministry of Health. [EU04]*

The inefficiency of the disaggregated nature of the European public health policy to respond to a health crisis became evident during the COVID-19 pandemic [27], where the European Commission provided competences of risk management to ECDC which capacity was previously excluded [28], something that historically differentiated ECDC from CDC [27,29]. For mosquito-borne diseases ECDC and CDC institutions released guidelines for risk assessment that are conceptually different. The ECDC requires a risk assessment based on mosquito suitability and presence, in addition to historical human risk [30], while the CDC recommends a certain vector index level to be above a threshold to trigger the response [31]. The structural differences in the constitution of EU and US [32] are reflected in the different approaches to surveillance by the public health stakeholders, with US interviewees mentioning the CDC guidelines explicitly, while the European interviewees relying on their respective national guidelines (that can still differ from country to country within the European Union):

*[We are] following our national infectious disease register […] and bringing cases regularly to our line list. And then from this line list we are communicating with the hospital districts and asking them to interview our patients because we, as I said, we do an enhanced surveillance. [EU05]*

*[the] mosquito recording scheme/ mosquito watch was set up in 2005 with the Chartered Institute of Environmental Health [and] heightened since 2016, since the Zika global outbreak, and we work with port health officers, so airports, seaports, inland border facilities and then with local authorities at those locations also, but then also on truck stops, motorway service stations. [EU07]*

*[In] our response programs related to West Nile, we closely follow the CDC's published guidelines and have a program which appropriately escalates a phased response based on the indications that we're getting from our laboratory testing. We get about a 48-h turnaround on submissions and results. So, that's a large part of our early warning system or early detection system […]. [US09]*

*I think having the CDC, the guidance, the unity of guidance and leadership […] advanced the way that we responded and collaborated. Ever since then that's been the standard in doing this sort of door-to-door response [Note: door to door inspections and abatements around the circumference of the reported case and based on the Ae. aegypti flight range] the reporting has been more immediate, more consistent and more organized. [US03]*

### 3.2 Lack of centralisation increases uncertainty in the application of mosquito surveillance and control guidance

A large degree of subjectivity is still left to the surveillance and control managers due to the lack of guidelines:

*We saw an area that looked like it was a pretty good habitat for mosquito [and we] trapped in that area. So really, our intention [was to] have a nice even distribution of traps. So that we could monitor the activity of the mosquitoes and where the virus was getting more active in the community. [US01]*

*We literally would go by ZIP code [that covers all our] county. […] we start learning about positive mosquito pools, we started getting them. […] All our positive pools are gonna be regular [routine] trap sites. [US05]*

*[…] a Vector Index of 200 is our trigger and go out and treat but we didn't believe that it was. […] Since we started this, all of our treatments are in the higher risk areas because our medium risk areas never hit that threshold. But basically it's going to be the neighbourhoods with the most people. [US02]*

*Earlier in the season, in the summer season, when things generally pick up for vector-borne diseases, we would try to rank the cities in terms of risk. There isn't a totally scientific way to rank them. We just go by number of cases sometimes or the incidence. [US03]*

*If we see numbers in our traps above our set thresholds we do Integrated Pest Management. [The threshold is] 5 numbers of Culex quinques or Aedes aegypti in areas. [US08]*

*Pathogens surveillance all over the year and the difference is that you start to have public health action when you have the virus in the vector. [EU09]*

Similar subjectivity surrounds when to intervene to reduce season transmission (or mosquito nuisance season). Due to its costs and number of personnel involved, mosquito surveillance and control interventions take place from late April - early May to September ‐ October [EU07, EU09, EU14, US01, US04, US08, US10, US11] or later for areas with sustained high temperatures and/or high rainfall [EU02, EU11, EU12, US05, US09], or all year long for certain areas [EU13, US02, US06, US07]. However, some authors suggested that it would be appropriate to focus the mosquito control activities late in the season and to target overwintering mosquitoes [33]. The current decisions are based on informal forecasting which is also prompted by the lack of epidemiological measures associated to entomological and climate conditions for determining ‘the right temporal window’ [34] for surveillanc and control:

*We do try to communicate with the vector control districts early. We have a pretty good relationship with them, so we chat over email and try to meet with them early in the year to get a sense of what the mosquito populations look like and such […]. So that's kind of our early warning system. [US03]*

*Surveillance is a big part of my organization […] but only in what we perceive as a transmission season. [EU11]*

*The levels of threat are set by conversations between [public health agencies, public laboratories and university]. They've been set at the same level for a while now. The calculations themselves are designed to be done biweekly, so it's two weeks. But the spatial scale is whatever you choose it to be. [US10]*

*We've transitioned away from [untargeted control and now] we rely on the mosquitoes to tell us what's going on real time through in-house PCR testing… We wanted to focus our resources in high-risk areas of course and spend less effort in the low risk areas where we typically don't have cases. [US02]*

*We have 30 trucks with Ultra Low Volume sprayers on the back, but we will not go out and do adulticide sprays until we start seeing that the vector index is in the range of our threshold for going out in spraying and at which point we will only spray in areas where we see a higher vector index. [US04]*

*[…] during the summer months, so during the West Nile virus season, blood donations are screened and then there is of course also surveillance in animals. [EU13]*

*We check daily the results of laboratory networks and we can catch up cases who have not been notified to regional offices. [EU12]*

The difficulties in applying generic guidelines (host, vectors and pathogens may be present in different quantities and forms – i.e. vector and pathogens hybrids) and the subjectivity introduced by public health managers and the risks associated with them [3], have raised calls for more centralised national strategies [35,36] in order to overcome the lack of technical capacities at local level, and facilitate the development of new control tools (vaccine), large scale intervention (national deployment of insecticide treatments) and surveillance tools (e.g. diagnostic tests). Only at national level it is possible to provide effective solutions and overcome the lack of economic and human resources [37].

*[…] basically, I'm the only person who is doing the surveillance of vector borne diseases and it depends from day to day, week to week, month to month. It depends, because of course, when there's a season for vector borne diseases, then it's different from compared to winter when there's nothing really happening. [EU05]*

*[…] then on top of that we also have to assign inspections and all these other complaints not just mosquito complaints but we get we get a bunch of bedbug complaints that come in so it's actually kind of difficult between a rotating staff of 2 to 4 staff members. We're not inspecting all the swimming pools and food establishments. [US05]*

*[…] sort out what's not a Culex versus what is, and get going, we simply do not have the time or the resources to have that sort of thing. [US04]*

*So this is like very complex and in this process is not only a matter of the digital systems [for surveillance of vector-borne diseases], but also we have to change the laws, you know. So, we are working at national level to have a better legal development. [EU08]*

*We had to change the law because we have a decentralised government. It was decided that for this particular group of invasive Aedes mosquitoes, the national government would take over the local government. [EU10]*

Finally, above any national strategy, multi-country interventions can be effective in the development of vaccines for mosquito-borne diseases [EU07], following the experience from COVID-19 [38]. At the moment this seems the most promising option for the elimination of mosquito- and other vector-borne diseases. Other methods currently trialled, such as sterile insect techniques [EU07] [39] are helping reduce insecticide use [EU10, EU14, US09], although insecticide use is still the most effective means to control vectors [US04, US10] [40].

### 3.3 Limited resources, limited modelling capabilities

Resources are one of the main issues faced by vector-borne disease surveillance and control agencies, although not everyone experiencing the same limitations, with large agencies better equipped in terms of resources and capabilities [25]. While the mandate of local surveillance for sub-national agencies is well accepted, national governments need to do more in terms of funding, resources, and support for research and control efforts.

Many interviewees reported using existing tools like commercial spreadsheet and GIS software or in-house tools to display their surveillance data [US01, US02, US04, US05, US08, US10, EU01, EU04, EU07, EU11]. The simple overlaying of mosquito data with human cases was viewed as beneficial to help identify triggers to initiate control [US02]. However, with limited resources, modelling capabilities are seen as luxury instead of an essential tool for successful surveillance and control [41]. Descriptive mapping and in-house analysis of the data were considered useful to support the understanding of risky habitat and confirming where anomalies or ‘odd’ catches happened [EU07]. Vector control agencies interrogate their data with basic descriptive statistics to provide an overview of what is going on [EU13]. This includes looking at averages over time to consider trends and to communicate with partners [US03]. Having the capacity in-house was viewed as beneficial with displays tailored directly to the needs of the group [US01, US10]. Despite the overwhelming adoption of mapping and visualization programs, moving from visualization to decision-making was viewed as a challenge. The lack of centralization also reduces the ability for those with modelling capacity to scale up or transfer their models because of incompatible data [26].

### 3.4 The way forward: Surveillance and control are community matters

Mapping mosquito densities, risk and interventions are recognized as a means to communicate vector control actions and promote transparency [EU07, US06]. Studies during the aftermath of mosquito-borne disease outbreaks have shown the importance of timely and consistent communication between experts and communities and, experts and policymakers to build trust in governmental health communications [42].

Some vector control agents worked as conduits between the data/science and the public, translating vector control data into information for the community [US01]. Often, this involved the use of social media [US01, US05], traditional media [US01, US03, US05, EU02, EU03, EU06, EU09, EU14] or on their own, locally managed websites [US01, US02, US03, US10, EU06, EU09, EU14]. Communication also involved their public health counterparts through data dashboards [US03, EU03, EU07] and through academic publications [EU03]. Improved acceptance of vector control's mission was one of the observed outcomes resulting from engaging and being transparent about vector control mission and decision-making with stakeholders and communities, as found in our study [US01] and by others [3].

*We have to be nimble in how we react, but very predictable in the things that we consider, you know. And I think that that has helped our community understand our approach. [US01]*

Engaging with the public outwardly was highly valued and has been shown to be critical in garnering support for mosquito control efforts, especially when testing or using novel techniques [43]. This can be achieved by pushing information, for example by taking the time to go into the community (e.g., farmer's markets, town hall/community events), and talking with decision makers and community members [US04]. Community engagement is also about soliciting information from the community – as for example to share opinion about pesticide application and to provide the opportunity to comment on adoption of industry best practices [US06]. Providing engagement opportunity is seen as positive in helping the communities understand the goals of mosquito control.

*I would say, 90 % of those people after we explain the way we make our decisions and the journey that we've been on in this program, appreciate the program as a whole. [Even the 10 % that disagree] have a lot more patience for the fact that we have done our due diligence with this using the best science that we have available and the integrated pest management approach. [US01]*

Integrating interventions is becoming a greener way of action, and often education is coupled with insecticide interventions [44,45]:

*Generally, if there was a lot of [WNV] cases in a certain area, they would do additional or targeted or enhanced education. Maybe they would put out notifications on their social media, and this is not the same across all the vector control districts [including] enhance vector control, vector abatement activities in the area. [US03]*

Aside from policy interventions, wider initiatives [46] are helping in the surveillance and control of mosquitoes. For example, VectorSurv in US is working to standardise surveillance methods, data collection and data management [47,48]:

*It's all integrated into VectorSurv. It's designed that vector control districts can just upload weekly there, all of their data, mosquito reports, dead birds, Sentinel chickens, and then he's worked with them so they have a very easy setup. […] It's become a really standardized system and has made it really easy to communicate from region to region to compare what's going on, because it's all standardized. […] so it is kind of a way to communicate to other states. [US10]*

*We do use [VectorSurv] to track where Aedes has been a concern. […] When detections have been, how often it has been. It is mostly to get a sense of the general level of risk, but not in a way that's very quantifiable like a certain criterion and then we can implement this sort of plan,* etc. *But more as a general qualitative way to assess what our risk has been. [US03]*

At community level, citizen-science-based apps in Europe [49,50] and US based crowd-sourced mosquito detection [51] are a valuable source of data of passive surveillance:

*We always do some media campaign to promote the website and to ask people to send pictures to website [or app]. I think last year we had about 300 photos. This year we have already 600. […] it's really successful because we had last year nine new locations [and] this year we have already 16. So, it's invasion… [EU06]*

Therefore, community science programs are growing in value and serve not only for dissemination and engagement but also to direct mosquito control activity and broaden the reach of vector surveillance activities. Other examples include the capacity for community members to upload photos with timestamps and location, to be followed up by the vector control agencies (e.g., EU06). This, at the same time, requires collaboration across disciplines with the desire of being a true cross-sectoral network actively exchanging information [EU05, EU08].

*I would say that the, the better connections and the better network you have with cross*-*sectional institutions and the more you increase the chance of using and taking advantage of the models and as such tools. [EU05]*

## 4 Limitations

The present study is affected by several limitations. First the sample size. Although there is a consensus that 15–30 interviews are sufficient for case studies [52], the heterogeneity in the structure and remits of public and animal health agencies within EU and US is large. In addition, the disease status can also vastly differ, with some regions experiencing emerging mosquito-borne diseases while others facing their established endemic transmission. Nonetheless, we found that similar issues were brought up repeatedly across interviews, suggesting that thematic saturation had been reached, with few agencies facing unique or novel challenges.

In this study, we considered mosquito-borne diseases, however, challenges in US and EU public and animal health come from other endemic vector-borne diseases too, such as tick-borne or sand-flies-borne [53], and therefore surveillance should account for the diversity of vector-borne diseases and integrate the interventions (see Conclusions) [54].

We did not focus on any specific zoonosis, although some are mentioned. In particular, West Nile Virus (WNV) typically circulates in a mosquito-bird enzootic cycle, with migratory birds playing a major role in introducing the virus into new regions. Humans and equines become infected through the bite of an infected female mosquito, particularly of the genus *Culex*. The importance of this disease for both humans and animals is dictated by the high fatality rate in humans and horses, although the impact appears to be lower in birds [55]. The multiple systems (human, animal, insect) involved in the transmission of WNV cannot, therefore, be addressed solely by public health agents, but require coordinated efforts across sectors, including veterinary and environmental health, who were not the subjects of our study.

## 5 Conclusion

In this work we described the continued barriers to attaining mosquito surveillance and control missions. Surveillance and control of vector-borne diseases should be based on an integrated system that takes into consideration optimisation of the surveillance design, interventions, communication, intersectoral collaboration and evaluation, given the complex dynamics of these disease systems (often involving multiple components such as the vector, animal host, human host and dead end hosts) [33].

In the seminal article dating back to 2011, Braks et al. [56] argue that vector-borne disease control intervention development needs the epidemiology of the disease to be fully understood and the intervention effectiveness measured. These are clearly two unreached objectives for many vector-borne diseases that are slowing down preparedness and their elimination in both endemic and emerging phases [57]. In fact, while surveillance can be seen as simply resource and capacity demanding, control has still some fundamental flaws as for example the absence of a generally accepted mosquito density threshold to guide decisions about the degree of vector population suppression that must be attained, or for how long this suppression must be maintained to reduce human disease transmission or nuisance [58]. Currently, public health agencies use a pragmatic approach based on coordinating surveillance activities and modify (improve) them based on field evidence.

Concern for the potential emergence, outbreak and spread of mosquito-borne diseases in and to EU and US has not been met with equivalent development of entomological experience, modelling capabilities or funding. There is significant investment in technical as well as infrastructural capacity, particularly in US (most of the agencies are well equipped for mosquito abatement) [58]. However, agencies are struggling in designing, developing or employing statistical models to achieve a more precise entomological forecast, which will allow more effective and cost-benefit appropriate decisions [59]. This lack of modelling capacity will seem even more anachronistic in the short term, thanks to the increased accessibility of customisable open foundation models [60]. To be effective, mosquito surveillance and control programs require a strong organisational base and transdisciplinary co-ordination between stakeholders, communities, skilled technicians, biostatistician/modellers and operators, appropriate equipment, and sufficient financial resources [33]. The ‘Global Vector Control Response 2017–2030’ by the WHO outlines a strategic, top-down framework for strengthening vector control globally, emphasising the need for strong country leadership, intersectoral coordination, and regulatory support [61]. However, the feasibility of such a top-down approach depends heavily on political will, governance capacity, and resource availability in each country. Integrated collaboration under a One Health framework is a strategy to overcome fragmented governance by promoting cross-sectoral coordination, shared surveillance systems, and joint response protocols through strengthening inter-agency coordination, data sharing, and integrated capacity (i.e. joint roles). The advantage of this strategy is exemplified by WNV disease, where integrated surveillance systems can detect WNV in birds and mosquitoes first before human cases emerge [23,62]. This strategy could be implemented through civic responsibility and community engagement (grassroots leadership) enhanced by education and group initiatives. The One Health approach is aimed at unifying fragmented systems, enhancing early warning capabilities, and promoting sustainable disease control which demands structural reforms, participatory governance, and sustained investment in cross-sectoral collaboration. This need is urgent given the current global trends, such as climate change, international travel and trade, and bioterrorism, that may increase the likelihood of outbreaks, emergence and spread of mosquito-borne diseases [63].

## CRediT authorship contribution statement

**Luigi Sedda:** Writing – review & editing, Writing – original draft, Visualization, Validation, Supervision, Software, Resources, Project administration, Methodology, Investigation, Funding acquisition, Formal analysis, Data curation, Conceptualization. **Elizabeth Wrench:** Writing – review & editing, Visualization, Validation, Software, Methodology, Formal analysis, Data curation. **Thomas C. Moore:** Validation, Methodology, Investigation, Formal analysis, Data curation. **Kaitlyn Wolfe:** Validation, Methodology, Investigation, Formal analysis, Data curation. **Julie-Anne A. Tangena:** Writing – review & editing, Validation, Supervision, Software, Methodology, Investigation, Formal analysis. **Heidi E. Brown:** Writing – review & editing, Writing – original draft, Visualization, Validation, Supervision, Resources, Methodology, Investigation, Funding acquisition, Formal analysis, Data curation, Conceptualization.

## Declaration of competing interest

The authors declare that they have no conflict of interest.

## Data Availability

The data that has been used is confidential.

## Appendix A Supplementary data

Supplementary material Image 1
