# Supplementary material for: Challenges in the surveillance and control of mosquito-borne diseases in Europe and United States. The perspective from public health experts
Source: One Health. 2025 Jul 8;21:101133. doi: 10.1016/j.onehlt.2025.101133 (PMC12275154; doi:10.1016/j.onehlt.2025.101133)
Supplement: Supplementary file 1 — Supplementary material [file mmc1.docx]

## Appendix A. Supplementary data

### Interviewee Contact Email

Dear ____,

My name is ____ and I am working on a joint research project with Lancaster University and the University of Arizona. Our team is investigating how vector control agencies optimize their vector surveillance and control designs in Europe as well as United States. This project is funded through Lancaster University’s Global Advancement Fund (GAF). With the information collected we will compile and compare vector control strategies in the United States and Europe to assess current practice and challenges experienced by health authorities. Additional information about the project is provided in the attached **leaflet**.

We are reaching out to you because of your [involvement in vector control in _____.]

If you have about 45 min, I would appreciate learning about how your institution deploys preparedness, surveillance and control plans for vector-borne diseases. We would like to schedule a meeting at your convenience, please let me know dates and times in the coming weeks that work for you. The meeting will be on Zoom or Teams, but please let us know if you have any preference for these or any other means of communication.

Thank you for your time.

Sincerely,

Signed

### Leaflet: creating operational research in vector-borne diseases

In most developing countries, vector-borne infections are a major public health challenge resulting in several million cases annually. However, recent changes in European, UK and US environments and climates have caused vector-borne disease to emerge and spread in the northern hemisphere.

With this project, we aim to lay the groundwork for US Federal and UKRI funded grants which incorporate two-way dialogue between academic and public health operators in the UK, Europe, and US. The project goal is prioritizing operational needs to control the emergence and spread of vector-borne diseases. Using lessons learned from our current projects in Africa and US, we will engage with public health agencies to identify potential solutions to operational gaps.

Using standardized methodologies, students for the project will interview regional public health leaders and integrate the information coming from our research in West Africa and the US BRACE projects. This GAF funding will involve Landcaster U campuses in Ghana and Germany to share results and promote a large network between UK, Europe, Africa, and US institutions.

### Questionnaire

#### Framing

Thank you for your time in speaking with me today. I am _NAME___ and I am {introduce yourself } working with Dr’s Brown and Sedda on this project.

We wanted to talk with you today because you are a coauthor on a manuscript or public health manager/head of division that uses VBD models and your affiliation is vector control. The goal of our project is to understand how models are being incorporated into West Nile virus or other emergent vector-borne diseases (Chikungunya) planning and control.

What flagged you for us was:

[INSERT CITATION/ REASON HERE]

We have about 13-20 questions (depending on if you count subparts), and if it is ok, I would like to record the audio for transcription purposes only – we will not directly identify you in any subsequent dissemination of findings. Once the transcript is produced the audio (video) file will be deleted. Would it be ok for me to start that now?

1. Can you tell me a little about your role in the public health institution?
   1. How do you organize the day-by-day vector intelligence or control activities?
   2. Do you post daily alerts or alerts with frequencies? Who are the recipients of these alerts?

#### Context Questions

These next questions are to help me understand what Vector Control looks like in your jurisdiction.

1. How long have you personally worked in vector control?

___ years

1. Would you say VC and planning for VC is a large part of your organization’s role? Are there dedicated “VC” departments/staff?
2. What are the kinds of activities your organization does?
   1. Spraying/ Larvicide / Surveillance trapping
   2. Frequency?
   3. Length of Season?
   4. Do you also issue alerts/ public messaging?
      1. If so, what triggers them?
3. Do you use any apps, databases, etc. Instead of paper records to collect and analyze surveillance data?
   1. If yes, can you tell me a little about those?

#### Main Questions

Thank you for helping me to understand vector control in your region. These next questions are specifically about the use of predictive modeling in planning vector control.

1. In what way do current advances in research and expert opinion influence your vector surveillance strategies?
2. To your knowledge, did the model get used to change any WNV control strategies?
   1. If not, do you have any thoughts as to why not?
3. Do you use/ work with modelers in your planning of vector surveillance?
   1. If yes, can you tell me a little about those?
      1. What kind of models?
      2. Who builds them (e.g., in-house, academic partners)?
         1. Was there a reason it isn’t done in-house?
4. If you work with models or modelers,
   1. Did working with modelers give you insight into how the data you collect is being used?
   2. Did working with modelers influence you to change the frequency or locations of trapping?
   3. Do models feed directly into the surveillance/control systems?
5. What do you think might increase the integration of modeling in VC programs?
   1. What facilitated/obstructed applying models to VC

#### Exploratory Questions

These final questions are again broader about vector control and the challenges to vector control.

1. What challenges do you experience when thinking about and planning for the vector control season?
   1. Can you describe a big success or a big failure you experienced working in VC?
      1. Do you think a model could have helped?
2. Do you have a ‘wish’ for when it comes to vector control?
   1. What time scale
   2. What spatial scale
   3. With what predictive accuracy? i.e., what do you want to predict?

#### Wrap-Up

I appreciate that you took the time to talk with me today to help me understand vector control from your perspective and the use of models in vector control.

1. Is there anything you wish I had asked, anything you want researchers to know about how vector control can be improved?
